# Conceptualizing Experimental Controls Using the Potential Outcomes Framework

**DOI:** 10.1080/00031305.2025.2554756

**Published:** 2025-10-16

**Authors:** Kristen B. Hunter, Kristen Koenig, Marie-Abèle Bind

**Affiliations:** a School of Mathematics and Statistics, University of New South Wales, Sydney, NSW, Australia; b Department of Molecular Biosciences, The University of Texas at Austin, Austin, TX; c Biostatistics Center, Massachusetts General Hospital, Boston, MA; d Department of Medicine, Harvard Medical School, Boston, MA

**Keywords:** Causal inference, Design of experiments, Experimental design, Negative control, Positive control

## Abstract

The goal of a controlled experiment is to remove unwanted variation when estimating the causal effect of the intervention. Experiments conducted in the basic sciences frequently achieve this goal using experimental controls, such as “negative” and “positive” controls, which are additional experimental components designed to detect systematic sources of variation. We introduce a taxonomy of clear, mathematically-precise definitions of experimental controls using the potential outcomes framework. These definitions are intended for pedagogical purposes: they may be used by educators to ensure that their students adhere to good statistical practice, and may also be useful for communication with practitioners who are less familiar with statistical concepts. We define three types of experimental controls based on assumptions about potential outcomes: treatment, outcome, and contrast controls. After each type of control is introduced, we provide examples of its use. We also discuss experimental controls as tools for researchers to use in designing experiments and detecting potential design flaws, such as identifying unwanted variation. We believe that experimental controls are powerful yet underutilized tools for reproducible, replicable, rigorous, and transparent research.

## 1. Introduction

### 1.1. Background and Motivation

The goal of a controlled experiment is to isolate the intervention as the sole causal mechanism influencing the outcome. An experimental control can help achieve this goal by helping to detect or remove systematic sources of variation from extraneous conditions (Kirk 1982). Experimental controls identify or reduce *unwanted* variation, better isolating the intended causal effect. Unwanted variation can be either systematic or random error, in statistical terms inducing either bias or variance. This work provides an accessible introduction to experimental controls for educators and practitioners, and illustrates definitions and uses of experimental controls in randomized experiments. The goal is to encourage good statistical practice, leading to the design of high-quality, reproducible experiments.

The most common use of the term “control” in a statistical study is a control group, which is a treatment group that receives a control treatment level, such as a placebo (Boring 1954). In this article, we use the term control to refer to an *experimental control*. An experimental control is an intentional set of experimental conditions that encodes a scientific assumption based on prior knowledge. In Section 2.2, we discuss the difference between a control group and an experimental control.

We believe researchers should include experimental controls in their arsenal of experimental design tools. Considering controls can deepen understanding of causal mechanisms, lead to more robust and reproducible conclusions, and help detect and prevent weak experimental designs. Due to unwanted variation, a poorly-controlled experiment may result in less precise estimates, or in the worst case, can result in misleading conclusions.

In this article, we provide an overview of experimental controls and some of their uses in experimental design. Our main contribution is to outline a taxonomy of clear, mathematically-precise definitions of different types of experimental controls using the potential outcomes framework (Splawa-Neyman, Dabrowska, and Speed 1923/1990; Rubin 1974). Our definitions align with how controls are commonly used in basic sciences. Our second contribution is to illustrate some practical uses of controls as diagnostic tools in experiments to identify and reduce systematic and random errors.

The definitions and examples in this article may be used to introduce students to fundamental concepts in the design of experiments. Statistics courses sometimes focus exclusively on the mathematical details of experiments, such as which type of factorial structure to use and how to properly estimate standard errors. Although understanding technical concepts is essential, we believe broader questions are sometimes overlooked, such as: Is the experiment addressing the intended question? Is it well-controlled?

Understanding experimental controls encourages students to think critically about the *quality* of experiments by highlighting their strengths and weaknesses. Our goal is for students to become practitioners who design high-quality, reproducible experiments and have a clear understanding of their limitations. In addition, these mathematically precise definitions can be useful for clear thinking and communication, and enhance interdisciplinary collaboration.

In current practice, there is a wide variation in the terminology and usage of experimental controls. In the basic sciences, the most commonly used terms are “negative control” or “positive control”, but these terms are not precisely defined and their use differs across studies. To provide some examples, the term “negative control” can refer to giving one group in an experiment no intervention (Fuenzalida and Ruegg 2019; Hassan et al. 2025), or giving one group the business-as-usual or current standard of care treatment (Shioda and Wakabayashi 2000; Zubaidah, Yuhana, and Widanarni 2015; Seriwatanachai et al. 2025). Alternatively, a negative control can be a separate population of units that should produce no response (Andrade et al. 2016). Similarly, in social science, the analogous concept of a “placebo test” can refer to a wide variety of checks on a study. A placebo test can involve: analyzing a different outcome assumed to not be associated with the treatment (Gläßel and Paula 2020), analyzing a different treatment assumed to not be associated with the outcome (Kogan, Lavertu, and Peskowitz 2016), or analyzing a different population assumed to not have a treatment effect (Ladd and Lenz 2009).

These examples show how different researchers can use the same term to refer to widely different study conditions, leading to confusion about the purpose and interpretation of experimental controls. Our definitions of experimental controls using simple mathematical equations based on potential outcomes are novel and could help ensure educators and practitioners are clear and precise in their use of these concepts.

### 1.2. Related Statistical Work

In this section, we provide an overview of some statistical uses of controls in epidemiology, political science, economics, and genetics.

A variety of methods which use negative controls in observational studies have been developed. Rosenbaum (2002, chap. 6) is one of the earlier works, and provides a discussion on using known effects to detect bias in observational studies. Vandenbroucke, Broadbent, and Pearce (2016) advocate for their use to strengthen causal conclusions by ruling out alternative explanations, such as in Keyes, Smith, and Susser (2014), although Sanderson, Macdonald-Wallis, and Smith (2018) suggest this approach be taken with caution. One of the most popular objectives is to help reduce confounding bias in observational studies (Alban et al. 2006; Flanders et al. 2011; Tchetgen Tchetgen 2014; Richardson et al. 2014; Sofer et al. 2016; Tchetgen Tchetgen et al. 2020; de Vries and Groenwold 2023; Miao et al. 2024; Piccininni and Stensrudd 2024). Shi, Miao, and Tchetgen Tchetgen (2020) provide an overview of these developments in epidemiology. Other uses of experimental controls in observational studies include sensitivity analysis for unmeasured bias (Rosenbaum 2021), detecting selection and measurement bias (Arnold et al. 2016), and creating a distribution of plausible effect estimates (Flanders et al. 2022).

A closely related concept to experimental controls is the placebo test, mostly used in political science and economics. Hartman and Hidalgo (2018) noted there was no clear consensus for the definition of a placebo test, and different authors may apply the term differently. Eggers, Tunon, and Dafoe (2024) promote the use of placebo tests, and define a placebo test as a “type of auxiliary analysis where the researcher checks for an association that should be absent if the assumptions underlying the design hold but might be present if those assumptions are violated in some relevant way.” They use directed acyclic graphs (DAGs) to outline a typology for different types of placebo tests, including placebo outcome tests, placebo treatment tests, and placebo population tests. Related concepts to placebo tests include “refutability” and “falsification tests”, which use empirical data to check if key assumptions do not hold (Angrist and Krueger 1999; Yang et al. 2014; Keele et al. 2019).

Finally, negative controls have also been used to deal with potentially noisy data in genetics. Gagnon-Bartsch and Speed (2012) use negative controls to correct for unwanted variation in microarray data, and a series of later developments known as RUV (removing unwanted variation) methods extends these methods to a wide variety of omics settings (Maksimovic et al. 2015; Trussart et al. 2020; Molania et al. 2021; Salim et al. 2022; Jang, Gagnon-Bartsch, and Speed 2023; Molania et al. 2023). Sanderson et al. (2021) apply the use of negative controls to genetic studies that use Mendelian randomization.

Our definitions most closely relate to the terms “negative control outcomes,” “negative control exposures,” “positive control outcomes,” and “positive control exposures” in previous statistical and epidemiological literature (Lipsitch, Tchetgen Tchetgen, and Cohen 2010; Tchetgen Tchetgen 2014; Shi, Miao, and Tchetgen Tchetgen 2020; Miao et al. 2024). Shi, Miao, and Tchetgen Tchetgen (2020) define them as follows. “A negative control outcome (NCO) is a variable known not to be causally affected by the treatment of interest. Likewise, a negative control exposure (NCE) is a variable known not to causally affect the outcome of interest.” For example, in examining the effect of air pollution on mortality, a negative control outcome is mortality on the day before the air pollution exposure, and a negative control exposure is air pollution level on the day after the mortality rate is measured (Miao et al. 2024).

We introduce our own terms and definitions that depart from these terms for a few reasons. First, Shi, Miao, and Tchetgen Tchetgen (2020) define these concepts mathematically, but in terms of conditional dependence and independence of random variables, which maybe opaque to applied researchers less mathematically inclined. Second, our work expands these concepts. We introduce two additional categories of experimental controls that do not fit into this existing framework. We also avoid using the terms “negative” and “positive” to emphasize that the mathematical definitions do not necessarily correspond to positive or negative mathematical quantities. For example, a negative causal effect could be a valid “positive control outcome.” By avoiding the terms “negative” and “positive”, we aim to reduce confusion and also avoid introducing conflicting definitions for the same term. Finally, we introduce the term contrast-control to emphasize an important concept. The previously defined terms do not make clear that they are making assumptions about causal *effects*, which are quantities that cannot be observed directly and must be estimated. In conclusion, we introduce new terminology and definitions both to provide more clarity and to distinguish from previously-used terms.

## 2. Motivating Example

### 2.1. Potential Outcomes Framework

As a motivating example, we consider the question: what is the short-term effect of drinking caffeinated coffee compared to drinking decaffeinated coffee on a person’s change in blood pressure from their own baseline level? We follow the hypothetical experiment detailed in Glass (2014) (Section Three: The Experiment). We will use this hypothetical example to illustrate different types of experimental controls using the potential outcomes framework. This section provides a brief introduction to the potential outcomes framework through this motivating example.

The random variable indicating treatment assignment (e.g., drinking caffeinated vs. decaffeinated coffee) for unit i is $W_{i}$. If $W_{i}=w_{at}$, unit i has been assigned to the active treatment $\left(at\right)$ level, and if $W_{i}=w_{ct}$, unit i has been assigned to the control treatment $\left(ct\right)$ level. Here, the active and control treatment levels are drinking caffeinated coffee and drinking decaffeinated coffee, respectively.

In an experiment, each unit has a different potential outcome given each possible treatment assignment. This framework is known as the Neyman-Rubin Causal Model (Splawa-Neyman, Dabrowska, and Speed 1923/1990; Holland 1986). The primary (p) outcome of interest, which we denote $Y_{i}^{p}$, is the change in blood pressure measured before and after an intervention. If unit i were to be assigned to the active treatment level $w_{at}$, we denote the potential outcome given treatment $w_{at}$ to be $Y_{i}^{p}\left(w_{at}\right)$. Similarly, if unit i were to be assigned to the control treatment level $w_{ct}$, we denote the potential outcome by $Y_{i}^{p}\left(w_{ct}\right)$. The fundamental problem of causal inference is that we can only observe one potential outcome per unit, because we cannot simultaneously assign more than one treatment level (Rubin 2008). We assume SUTVA, which refers to the stable unit treatment value assumption (Rubin 1980), and we assume the finite sample setting (Ding and Li 2018; Miratrix, Weiss, and Henderson 2021). We will expand the framework to allow for additional outcomes and treatment levels, which will be defined in Section 3. For now, let $Y_{i}^{k}\left(w_{j}\right)$ denote the potential outcome k for unit i had unit i been exposed to treatment level $w_{j}$.

The unit-level causal effect of primary interest is the average treatment effect (ATE)

$$\tau_{i}^{p}=Y_{i}^{p}\left(w_{at}\right)-Y_{i}^{p}\left(w_{ct}\right).$$

Given N units, our causal estimand of primary interest is

$$\tau^{p}=\frac{1}{N}\sum_{i=1}^{N}\tau_{i}^{p}=\frac{1}{N}\sum_{i=1}^{N}\left(Y_{i}^{p}\left(w_{at}\right)-Y_{i}^{p}\left(w_{ct}\right)\right).$$

In our hypothetical example, $\tau^{p}$ is the average difference between the blood pressure potential outcomes given the active treatment level of drinking caffeinated coffee and the control treatment level of drinking decaffeinated coffee. The analyst may also consider causal estimands of secondary interest, which can be used for descriptive purposes or for validation; we will discuss some potential secondary estimands when we introduce contrast-controls in Section 3.3.

### 2.2. Control Treatment Levels

Control treatment levels are essential to make a causal estimand well-defined. We discuss them here to elucidate their difference from the treatment-controls we will next define in this article. A control treatment level is a treatment level we choose that we would like to compare to the active treatment level. As stated in Rubin (2008), “a causal effect is, by definition, a comparison of treatment and control potential outcomes on a common set of units.” In our example, what is the effect of drinking caffeinated coffee on blood pressure *compared to* decaffeinated coffee? The control treatment level, decaffeinated coffee, is essential to define the estimand. Without the control treatment level, we would not have an estimand, because we would have a single potential outcome, and thus we would not be asking a causal question.

The choice of control treatment level isolates the particular effect of interest (Feller et al. 2016). For example, consider possible control treatment levels and their corresponding estimands in Table 1, which continue to follow Glass (2014, chap. 23). We have chosen $w_{ct3}$, decaffeinated coffee, as a control treatment level, which focuses on just the isolated effect of the caffeine in coffee. Alternatively, choosing water $\left(w_{ct1}\right)$ would expand our estimand to include the effect of all the non-fluid components of coffee, such as any other compounds in coffee in addition to caffeine.

## 3. Experimental Control Definitions

We define our taxonomy of experimental controls with two axes: first, whether they are null controls or non-null controls; and second, whether they are treatment, outcome, or contrast-controls. The first axis pertains to whether the potential outcomes (or their contrast) are assumed to be zero or nonzero, while the second relates to which additional potential outcome(s) we are making an assumption about.

Table 2 contains a summary of the control definitions on these axes. We will walk through the definition and examples for each type of control.

### 3.1. Treatment-Controls

#### *Definition 3.1* (null treatment-control).

A null treatment-control is a treatment level $w_{nt}$ for which the primary potential $Y_{i}^{p}$ outcome is assumed to be zero for unit i:

$$Y_{i}^{p}\left(w_{nt}\right)=0.$$

#### *Example 3.1* (caffeine experiment).

No intervention (i.e., remaining quietly seated) is a null treatment-control on change in blood pressure. If no intervention occurs, the true latent blood pressure of a person should not change.

#### *Example 3.2* (neuromodulation techniques).

Rezaei and Khanmohammadi (2025) investigated the effect of noninvasive neuromodulation techniques transcranial direct current stimulation (tDCS) and neurofeedback (NFB), which are brain stimulation techniques.

##### Outcome(s):

the primary outcome was short-term change in motor learning $\left(Y_{i}^{p}\right)$, measured by change in reaction time, change in response accuracy, and change in skill index.

##### Treatment and treatment levels:

the researchers explored two treatments, tDCS and NFB, with three treatment levels each. For example, tDCS $\left(W_{i}\right)$ had treatment levels: real tDCS, in which the researchers placed electrodes on the participant and ran a current between them (active treatment level, $w_{at}$); sham tDCS, in which the researchers placed electrodes on the participant but did not run a current between them (placebo treatment level, $w_{ct}$); and no intervention (null treatment-control level, $w_{nt}$).

For each neuromodulation technique, the study had an active treatment level and a placebo treatment level to evaluate the magnitude of any placebo effect. In addition, there was also a treatment level of no intervention, which the researchers called a “passive control.” The study was conducted in healthy individuals, and no short-term change in motor learning was expected with the passive control of no intervention. Here, the “no intervention” level was a null treatment-control, because the outcome (a change score) is assumed to be zero. However, some people in the null treatment-control group did show a significant change in response accuracy. This observed change indicated that any change in response accuracy due to the treatments of interest should be cautiously interpreted.

##### Purpose of control:

A departure from the control assumption implied response accuracy may have been changing due to other factors besides neuromodulation, the treatment of interest.

Conceptually, the definition of a null treatment-control is that it is a treatment resulting in a null potential outcome. In a different context, a null outcome may not be 0; for example, if the outcome is a ratio of blood pressure measurements, the null treatment-control assumption would be that the outcome is 1 rather than 0.

When choosing the control treatment level, which is the comparison treatment, the researcher could choose a null treatment-control (Section 2.2). However, often the null treatment-control is not the ideal choice because it may not be the most useful as a point of comparison. For example, comparing caffeinated coffee to no intervention (a null treatment-control) gives an estimate of all components of coffee, and would not isolate caffeine alone. Drinking fluids can temporarily increase blood pressure in some people (Ma et al. 2019), so by not comparing coffee to another fluid, we estimate the combination of caffeine, other components in coffee, and the fluid. Instead, a common choice of control treatment level is a “placebo” treatment. Placebo treatments are often expected to have a nonzero effect on the outcome, so they are not null treatment-controls.

#### *Definition 3.2* (non-null treatment-control).

A non-null treatment-control is a treatment level $w_{nnt}$ for which the primary potential outcome $Y_{i}^{p}$ is assumed to be nonzero for unit i:

$$Y_{i}^{p}\left(w_{nnt}\right)\neq 0.$$

#### *Example 3.3* (caffeine experiment).

Taking a hypertensive drug (e.g., a drug that raises blood pressure) is a non-null treatment-control on change in blood pressure. If a participant is given a hypertensive drug that is *a priori* known to be effective for that participant, their underlying blood pressure should increase.

#### *Example 3.4* (drug discovery).

Kwasny and Opperman (2010) outline a screening procedure for identifying inhibitors of biofilm for pathogenic bacteria. Biofilm is a protective structure that encases a bacterial community and can cause the bacteria to be more resistant to antibiotics.

##### Outcome(s):

the primary outcome is the level of biofilm growth on an assay plate $\left(Y_{i}^{p}\right)$.

##### Treatment and treatment levels:

the treatment is the inoculation of each assay plate $\left(W_{i}\right)$, with treatment levels: a compound which is a potential biofilm inhibitor (active treatment level, $w_{at}$), a known effective antibiotic (non-null treatment-control level, $w_{nnt}$), and no compound (control treatment level, $w_{ct}$). The goal of the experiment is to evaluate which compounds successfully prevent biofilm growth. Each assay plate contains multiple wells, which may receive different treatment levels. The non-null treatment-control level, which the researchers refer to as a “positive control,” is an antibiotic known to cause 100% biofilm inhibition. This experiment also features a null treatment-control: the control treatment level, applying no compound to the plate, should cause 0% biofilm inhibition.

##### Purpose of control:

The two treatment-controls ensure the experimental conditions are as expected. The null treatment-control checks that biofilm develops without the test compound. The non-null treatment-control checks that biofilm inhibition is possible. If either the null treatment-control or non-null treatment control do not perform as expected, the results for the treatment of interest may not be reliable.

### 3.2. Outcome-Controls

#### *Definition 3.3* (null outcome-control).

A null outcome-control is an outcome $Y_{i}^{no}$ that is assumed to be zero for unit i given the active treatment level $w_{at}$:

$$Y_{i}^{no}(w_{at})=0.$$

We emphasize that $Y_{i}^{no}$ is a distinct outcome from the primary outcome of interest $Y_{i}^{p}$.

#### *Example 3.5* (caffeine experiment).

Change in body flexibility is a null outcome-control for drinking caffeinated coffee. The current scientific understanding is that body flexibility does not change after drinking caffeinated coffee.

#### *Example 3.6* (low-tech learning).

In Angrist, Bergman, and Matsheng (2022), researchers ran an experiment to evaluate whether sending educational text messages during school closures results in student learning, compared to no intervention.

##### Outcome(s):

the primary outcome was change in numeracy skills before and after the experiment $\left(Y_{i}^{p}\right)$. A secondary outcome was change in effort $\left(Y_{i}^{no}\right)$.

##### Treatment and treatment levels:

the treatment was educational text messages $\left(W_{i}\right)$, with two treatment levels: text messages (active treatment level, $w_{at}$) and no intervention (control treatment level, $w_{ct}$).

The researchers hypothesized that the text messages could have two potential mechanisms for causing an increase in a child’s numeracy skills. First, the intervention could cause a gain in cognitive skills by imparting specific knowledge. Second, the intervention could cause the child to increase the amount of effort put into learning, known as an “effort effect”, without imparting any specific knowledge. The researchers hypothesized that the intervention did not increase effort, but only increased cognitive skill, and wanted to investigate whether the effort effect was likely at play. Thus, they investigated a secondary outcome, the change in a student’s effort $\left(Y_{i}^{no}\right)$. The researchers measured effort at the beginning and end of the experiment by asking students a question that required primarily effort, and seeing whether students answered the question successfully. If more students answered this effort question successfully after the study than before, then the increase in numeracy skills may in part be due to an increase in effort. Here, effort change score is a null outcome-control: they expect no change in effort, a secondary outcome, due to the active treatment level of receiving text messages. The researchers indeed reported no substantial changes in effort in any of the treatment levels. They concluded that the increase in skill found in the study was likely due to a cognitive skill increase rather than effort.

##### Purpose of control:

The null hypothesis was that a change in effort did not cause the increase in numeracy skills; rather, the increase was due to an increase in cognitive skills alone. Based on the experimental control evidence, they cannot reject the null hypothesis. Using the experimental control, the researchers did not find statistical evidence that the increase in numeracy skills was due to an increase in effort.

#### *Definition 3.4* (non-null outcome-control).

Anon-null outcome-control is an outcome $Y_{i}^{nno}$ that is assumed to be nonzero for unit i given the active treatment level $w_{at}$:

$$Y_{i}^{nno}\left(w_{at}\right)\neq 0.$$

#### *Example 3.7* (caffeine experiment).

Change in electrolyte concentration is a non-null outcome-control for drinking caffeinated coffee. Electrolyte concentration is known to change after drinking caffeinated coffee.

#### *Example 3.8* (responders).

In Nguyen-Thu et al. (2018), researchers investigate cisplatin, which is a treatment for breast cancer. Some patients highly respond to cisplatin (responders), while others see little to no reduction in tumor size (non-responders). The researchers used a mouse model to find criteria to successfully predict early in the cancer treatment regime whether a mouse would be a responder or non-responder to cisplatin. The change in metabolic tumor volume (MTV) between day 0 and day 7 was found to be a predictor of responder status. In the following, we imagine a hypothetical follow-up experiment evaluating the effectiveness of two different cisplatin treatment regimes on breast cancer remission.

##### Outcome(s):

the primary outcome $Y_{i}^{p}$ is the survival time of the mouse. The secondary outcome $Y_{i}^{nno}$ is change in MTV between day 0 and 7.

##### Treatment and treatment levels:

the treatment is cisplatin treatment $\left(W_{i}\right)$, with two treatment levels: the standard of care regime (control treatment level, $w_{ct}$), and a new proposed treatment regime (active treatment level, $w_{at}$).

Both treatment levels will only be effective for responders, so the researchers want to screen for responder status. Change in MTV is a non-null outcome-control for responders: for responders, we expect to see a decrease in MTV.

##### Purpose of control:

The non-null outcome control is used to screen units for their responder status.

#### *Example 3.9* (attention checks).

In survey experiments, researchers often want to screen out inattentive or careless participants. Inattentive participants may not fully receive the intended treatment, and thus estimates of treatment effects will be biased toward zero (Kane, Velez, and Barabas 2023). One tool for removing these participants is attention checks, which are questions with known answers that indicate whether an individual is paying attention (Muszyński 2023). Dahlum, Pinckney, and Wig (2023) is one example of a survey experiment that used attention checks. The researchers investigated support for nonviolent protests. The survey gave participants different vignettes about hypothetical protests with specific characteristics, such as whether the hypothetical opposition was violent or nonviolent, and how the hypothetical government responded to protests.

##### Outcome(s):

the primary outcome was the individual’s response to whether they support a hypothetical opposition movement ($Y_{i}^{p}$). A secondary outcome was a binary variable corresponding to whether the survey participant answered an attention check question correctly ($Y_{i}^{nno}$), that is, $Y_{i}^{nno}=1$ if their answer is correct, and $Y_{i}^{nno}=0$ otherwise.

##### Treatment and treatment levels:

the experiment was factorial. Each factor j corresponded to a type of change in a vignette about hypothetical protests supplied to participants $\left(W_{ij}\right)$. There were at least two levels for each factor. For example, one factor was how the hypothetical government responded to protests, with two levels: a violent government response (active level, $w_{at}$), or a nonviolent government response (control level, $w_{ct}$).

The researchers added an attention check question ($Y_{i}^{nno}$) that asked participants to recall a detail from the vignette. An attention check is a non-null outcome-control: it is an additional outcome the researchers measured, with a certain assumed value. Here, $Y_{i}^{nno}$ is a binary variable corresponding to whether or not the participant answers correctly or not. All participants should get the detail correct (e.g., $Y_{i}^{nno}=1$) if they are paying attention to the vignette.

In this case, we expect the participants to get the detail correct regardless of what treatment level the participant is assigned to, so we expect $Y_{i}^{nno}\left(w_{at}\right)=1$ and $Y_{i}^{nno}\left(w_{ct}\right)=1$. Although we define a non-null outcome-control only given the active treatment level, $Y_{i}^{nno}\left(w_{at}\right)=1$, it would be straightforward to expand the framework to include an additional check for $Y_{i}^{nno}\left(w_{ct}\right)=1$.

##### Purpose of control:

If the participant passes the attention check, the participant is in some sense “complying” with the treatment, and we can more confidently assume that the treatment had an effect on the final survey response. The researchers used the attention check as an inclusion criterion and removed the participants who failed the attention check from their analysis. Note that this approach may be problematic if there is differential attention between treatment groups (Frangakis and Rubin 2002).

In many settings, applying an outcome-control is most natural if the experiment measures a change score. The fact that outcome controls most easily apply to change scores is somewhat limiting, but change scores are a very common experimental measurement across a variety of fields, including education, social science, and medical experiments (including Examples 3.2, 3.6, and 3.8). In other settings, an outcome control may be applied more generally. In Example 3.9, the non-null outcome-control is not a change score, although it is a binary variable. There are likely other contexts where outcome-controls are useful beyond change scores.

In using any type of experimental controls, the control is encoding an assumption; in other words, it is canonicalizing what you *assume* you will see. However, the true power of controls becomes most apparent when reality departs from your assumption. For example, in Example 3.2, the null treatment-control did result in a non-null outcome, which implied care should be taken in attributing causality to the treatment of interest. In Example 3.9, if the non-null outcome-control is instead null, for example, the participant fails the attention check, then caution should be taken in using that participant’s responses. The experimental controls in these cases exposed potentially flawed observations or experimental conditions.

### 3.3. Contrast-Controls

#### *Definition 3.5* (null contrast-control).

A null contrast-control is an effect $\tau_{i}^{nc}$ that is assumed to be zero for unit i:

$$\tau_{i}^{nc}=Y_{i}^{nc}\left(w_{atc}\right)-Y_{i}^{nc}\left(w_{ctc}\right)=0.$$

It is defined as a contrast of potential outcomes between an active treatment level $w_{atc}$ and a control treatment level $w_{ctc}$, where the final “c” stands for contrast.

#### *Example 3.10* (caffeine experiment).

The effect of caffeinated coffee compared to decaffeinated coffee on short-term change in body weight is a null contrast-control.

#### *Example 3.11* (voter turnout).

In Gerber, Green, and Shachar (2003), the authors asked: is voting habit-forming, that is, does voting in an election make someone more likely to vote in the next election? The researchers used an encouragement design to encourage voting in a 1998 election and looked at the downstream effect of voting in a 1999 election.

##### Outcome(s):

the primary outcome was whether someone voted in the 1999 election $\left(Y_{i}^{p}\right)$. A secondary outcome was wheter someone voted in the 1996 election $\left(Y_{i}^{nc}\right)$.

##### Treatment and treatment levels:

the treatment was voter encouragement $\left(W_{i}\right)$, with three treatment levels: personal canvasing (active treatment level, $w_{at1}$), direct mail (active treatment level, $w_{at2}$), or no intervention (control treatment level, $w_{ct}$).

The authors considered whether it is possible “some kind of accident of randomization occurred” that put more long-standing voters in the active treatment groups, which would make their treatment seem beneficial when it was in fact due to underlying covariate imbalance. To check, they looked at voter turnout in 1996 $\left(Y_{i}^{nc}\right)$, the previous election, before the experiment occurred. There can be no true causal effect of an intervention in 1998 on voter turnout in 1996. Therefore, comparing voter turnout in 1996 in an active treatment level group to turnout in the control treatment level group is a null contrast-control. The researchers found no substantial difference in voter turnout in 1996 between the different treatment groups.

##### Purpose of control:

The control assumption held, which implies any difference in voter turnout was due to the treatment rather than due to underlying background differences in the treatment groups.

Generally, when choosing a contrast-control, we have two choices. One option is to keep the primary outcome, so we set $Y_{i}^{nc}=Y_{i}^{p}$, and choose at least one secondary treatment level $w_{atc}$ or $w_{ctc}$. The second option is to keep the primary treatment levels, so we set $w_{atc}=w_{at}$ and $w_{ctc}=w_{ct}$ and choose a secondary outcome $Y_{i}^{nc}$. In Example 3.10, we chose a secondary outcome (short-term change in body weight, $Y_{i}^{nc}$), the primary active treatment level (caffeinated coffee, $w_{at}$), and the primary control treatment level (decaffeinated coffee, $w_{ct}$. Similarly, in Example 3.11, we chose a secondary outcome (voter turnout in 1996) while keeping the primary treatment levels. Alternatively, an example of changing the treatment levels is the effect of caffeinated coffee (primary active treatment level $w_{atc}$) compared to caffeinated water (alternative control treatment level, $w_{ctc}$) on change in blood pressure (primary outcome, $Y_{i}^{p}$). This effect is a valid null contrast-control only if we believe from prior evidence that the only component of coffee that affects blood pressure is caffeine.

#### *Definition 3.6* (non-null contrast-control).

A non-null contrast-control is an effect $\tau_{i}^{nnc}$ that is assumed to be nonzero for unit i:

$$\tau_{i}^{nnc}=Y_{i}^{nnc}\left(w_{atc}\right)-Y_{i}^{nnc}\left(w_{ctc}\right)\neq 0.$$

#### *Example 3.12* (caffeine experiment).

The effect of caffeinated coffee compared to decaffeinated coffee on change in reaction time is a non-null contrast-control (Santos et al. 2014).

#### *Example 3.13* (gingivitis).

In Lorenz et al. (2006), researchers ran an experiment to determine the effectiveness of a new mouthrinse on gingivitis. The previous standard-of-care mouthrinse contained alcohol, and the authors investigated whether two new mouthrinses that did not contain alcohol were as effective.

##### Outcome(s):

the primary outcome was four variables measuring gingivitis presence and severity $\left(Y_{i}^{p}\right)$.

##### Treatment and treatment levels:

the treatment was oral health habits $\left(W_{i}\right)$, with four treatment levels: test mouthrinse 1 (active treatment level, $w_{at1}$), test mouthrinse 2 (active treatment level, $w_{at2}$), standard mouthrinse (“positive control”, $w_{atc}$) or placebo (control treatment level, $w_{ct}$).

The main causal estimands were the mean differences in gingivitis between each active treatment level (new mouthrinse) and the control treatment level (placebo). In addition to their main estimands, the researchers considered a secondary estimand: the mean causal effect on gingivitis of the new mouthrinse $\left(w_{at1}\right)$ compared to the standard of care $\left(w_{atc}\right)$, an existing mouthrinse containing alcohol known to be effective in preventing gingivitis. This effect is a non-null contrast control, where they chose an alternative treatment level, but measured the primary outcome. The researchers used the non-null contrast-control as a standard of comparison to better evaluate the effect size of the new mouthrinse: was it a similar magnitude to the current standard of care? An effect may not be of clinical interest if the estimated effect size is much smaller than the standard of care, even if it is “statistically significant.”

##### Purpose of control:

The non-null contrast-control was used as a standard of comparison to determine clinical relevance. The authors found that the new mouthrinses without alcohol had a similar estimated effect size compared to the “gold standard” with alcohol, so they conclude alcohol is not a necessary ingredient.

#### *Example 3.14* (fact-checking).

In Nyhan and Reifler (2015), the authors ran an experiment to estimate the effect of fact-checking on the accuracy of state legislators’ public statements.

##### Outcome(s):

the primary outcome was how many false statements a politician made during their re-election campaign $\left(Y_{i}^{p}\right)$.

##### Treatment and treatment levels:

the treatment was the fact-checking policy $\left(W_{i}\right)$, with three treatment levels: warning letter to a politician (active treatment level, $w_{at}$), no letter (control treatment level, $w_{ct}$), or neutral letter (Hawthorne treatment level, $w_{atc}$).

The active treatment level was a warning letter to legislators that “emphasized the risks of having misleading or inaccurate statements exposed by fact-checkers” (Nyhan and Reifler 2015). The authors had an additional treatment level, which we will call the Hawthorne treatment, which was a letter that “alerted legislators that we were conducting a study of the accuracy of statements made by politicians,” but did not contain any information about the dangers of false statements (Nyhan and Reifler 2015). This treatment level was designed to counteract a phenomenon known in political science as the “Hawthorne effect,” that study participants may behave differently when they know they are being studied. The effect on false statements of the Hawthorne letter $\left(w_{atc}\right)$ compared to no letter $\left(w_{ct}\right)$ was a non-null contrast control, where they chose an alternative treatment level, but measured the primary outcome. The researchers added this estimand because they wanted to compare the magnitude of the effect size for their active treatment level to the Hawthorne letter.

##### Purpose of control:

The non-null contrast-control was used to determine if any observed treatment effect was due solely to the units being observed, or whether there was an additional effect above the Hawthorne effect. The researchers found a significant reduction in false statements in the active treatment group compared to the other treatment groups. They found no significant difference between the Hawthorne letter and no letter. Using the experimental control, the researchers did not find evidence that the observed decrease in false statements was due to the Hawthorne effect.

One unique aspect of contrast-controls is that they can never fully be observed directly: they must always be estimated. Just as with any causal effect, they are defined on a pair of potential outcomes, only one of which can be observed at a time. Treatment-controls and outcome-controls can be observed directly. After the contrast-control effect is estimated, then contrast-controls are used similarly to other types of controls.

Just like for treatment-controls and outcome-controls, the estimated contrast-control may or may not match the assumption that was originally made. A mismatch between the estimated and assumed contrast-control indicates a potential problem in the experiment. In Example 3.11, although there can never be a *true* causal effect of get-out-the-vote methods in 1998 on voter turnout in 1996, we could see an *estimated* causal effect. If there was a non-null estimated effect, it would indicate an experimental flaw, in this case most likely imbalance in voter characteristics across treatment groups.

## 4. Using Controls in Experimental Design

One of the most important uses of an experimental control is as a diagnostic tool to detect potential experimental flaws. A failed diagnostic test means the experiment may not be isolating the effect of interest. We now discuss more examples of how experimental controls can be important components of a researcher’s toolbox to detect such flaws.

### 4.1. Experiment Stage

Experimental controls can be incorporated at a variety of stages of an experiment: a pilot study, the pretrial stage (in which the same units used in the experiment have measurements collected before the experiment begins), the main experiment, or a reproduction study. The best stage to incorporate experimental controls depends on the cost and feasibility of replicating the study.

Ideally, a problematic control diagnostic test would result in the researcher re-designing and repeating the experiment. For example, a small-scale biological experiment on fruit flies may be easily repeatable, with adjustments in each repetition based on the experimental control diagnostics. If a control diagnostic test reveals a systematic measurement error, the measurement tool can be re-calibrated before repeating the experiment. Thus, experimental controls can be used during the main stage of a study for easily replicable experiments.

In many fields, experiments are often difficult to replicate due to cost or feasibility, such as with expensive interventions like new education or economic programs. For studies that are difficult to replicate, we recommend that, if feasible, researchers run a small pilot study in which they measure as many controls as possible. During a pilot study, the full experiment is conducted on a smaller set of units, usually not the same ones that will be in the main experiment. The cost of running a pilot study may well be worth the benefit of detecting flaws before the full experiment, rather than uncovering flaws after the fact.

Experimental controls can also be used after an experiment, during a reproduction study, to evaluate the quality of the results. Many fields, from psychology to medicine, have suffered from a replicability and reproducibility crisis. Hilgard (2021) propose using a “maximal positive control” (by our definitions, a maximal non-null treatment-control) to evaluate the quality and reproducibility of previous studies. A maximal non-null treatment-control is a treatment level chosen to elicit the largest possible effect size on a given measure. If a study’s reported effect size exceeds the maximal non-null treatment-control, then the result is suspect.

#### *Example 4.1* (violence and video games).

Hilgard (2021) give an example of how a maximal non-null treatment-control could be used in an experiment designed to measure the level of aggressive thoughts caused by a violent video game. Although the video game experiment is hypothetical in this case, the example is inspired by a previous experiment that found a suspiciously large effect size for the effect of playing a violent video game on aggressive thoughts, which the authors believe may not be reproducible.

##### Outcome(s):

the level of aggressive thoughts is measured through a word completion task. Participants are given word stems like KI_—_, which can be completed with words such as KISS, KIND, or KILL. The primary outcome is the number of aggressive words used in the word completion task $\left(Y_{i}^{p}\right)$.

##### Treatment and treatment levels:

the treatment was the activities and instructions before trying the word completion task $\left(W_{i}\right)$. There are three treatment levels. The two main treatment levels are playing a violent video game and receiving no further instruction (active treatment level, $w_{at}$), and completing a crossword puzzle and receiving no further instructions (control treatment level $w_{ct}$). The author propose an alternative treatment level as a maximal non-null treatment-control: the participants do no activity before the word completion game and then are instructed to try the word completion task with as many aggressive words as possible.

##### Purpose of control:

Hilgard (2021) state the estimated effect size for playing video games would only be plausible if it is smaller than for the maximal non-null treatment control. The experimental control allows evaluation of whether the estimated effect size is realistic, or a potential sign of an unreproducible experiment.

Although Hilgard (2021) use maximal non-null treatment controls in reproduction studies, a researcher may want to proactively incorporate a maximal non-null treatment control into their study, or even a pilot study, as their own quality check.

In the supplementary materials, we discuss other examples of using controls during different experiment stages, using the hypothetical caffeine experiment. A non-null treatment-control can be used during a pilot study to determine optimal timing. Controls during a pretrial phase can be used to identify important subpopulations, such as responders or compliers. A null treatment-control can be used during the main stage in settings with measurement error.

### 4.2. Identifying Unwanted Factors

In an ideal, well-controlled experiment, the only component that *should* influence the outcome is the given treatment level. Unwanted factors can result in unwanted variation in the observed outcome. Here, we use the term unwanted factor to refer to an experimental condition which influences the outcome, but which is not intentionally manipulated by the researcher. In this section, we discuss using experimental controls to identify unwanted factors. First, we return to the hypothetical caffeine experiment. Second, we give another example of a political science experiment with unwanted factors.

A null treatment-control is one tool to help us detect whether the experiment was poorly controlled. Assume the experiment protocol is the following: researchers measure the study participants’ blood pressure, give a certain treatment, put participants in a waiting room for 20 minutes, and measure their blood pressure again. Imagine there are two waiting rooms. The first room is quiet, with gardening magazines to pass the time, and the second room has a TV showing live updates of election results. Some participants in the second waiting room may experience stress and a corresponding increase in their blood pressure. In this scenario, blood pressure is being influenced by an unwanted factor, the potential stress of the election results. A diagnostic test using a null treatment-control can help the researcher assess whether unwanted factors may be influencing the outcome.

To implement the diagnostic, we use three intervention groups: active treatment level, control treatment level, and null treatment-control level (no intervention). The waiting room variation will result in unwanted variation in blood pressure for all three groups. We can potentially detect this unwanted variation using the null treatment-control group. In a well-controlled study, units in the null treatment-control group should have an average of zero for the potential outcome of short-term change in blood pressure. Thus, we can calculate this average change as a diagnostic statistic. In the stressful waiting room setting, we find this average is positive and significant. After a failed diagnostic, researchers should conduct an in-depth examination of experimental conditions. If the average change were instead not substantially different from zero, we would have more confidence of no unwanted factors.

Unfortunately, an experimental control cannot fix a poorly designed experiment after the fact, but only provides a means of detecting the flaw. This limitation is why we recommend that researchers employ experimental controls during a pilot phase to detect poor experimental conditions in advance.

Is detecting this experimental mishap important? If the waiting room assignment is independent from the treatment assignment, the effect of stress will be balanced on average across the active treatment and control treatment groups, so the estimated treatment effect would still be unbiased. In this case, the unwanted factor is not a confounder. However, the estimate could suffer from high variance, and thus low power to detect a treatment effect.

On the other hand, if the waiting room assignment is influenced by the treatment assignment, then the unwanted factor is a confounder, and the estimated treatment effect would be biased. Perhaps the researchers had two coffee machines, one for caffeinated coffee and one for decaffeinated coffee, and tended to place participants in the waiting room closer to the coffee machine used for that participant. This confounding would result in a difference in blood pressure change between active treatment participants and control treatment participants that is unrelated to the treatment.

This hypothetical example of confounding between treatment assignment and the unwanted factor may seem contrived, but these settings do occur in experimental settings. To give one example, survey experiments on political beliefs are prone to unobserved common causes between the treatment and outcome (Dafoe, Zhang, and Caughey 2018).

#### *Example 4.2* (political beliefs).

Dafoe, Zhang, and Caughey (2018) conducted an experiment inspired by Tomz and Weeks (2013), in which the authors ran a survey experiment relating people’s beliefs about democracy and military force.

##### Outcome(s):

the primary outcome is the respondent’s support for military force against a hypothetical country $\left(Y_{i}^{p}\right)$. Secondary outcomes $\left(Y_{i}^{nc}\right)$ are respondent’s beliefs about other characteristics of the hypothetical country, including the GDP and demographic characteristics.

##### Treatment and treatment levels:

the treatment is the characteristics of a hypothetical country $\left(W_{i}\right)$, with treatment levels: the country is a democracy (active treatment level, $w_{at}$), or the country is a non-democracy (control treatment level, $w_{ct}$).

Rather than asking about people’s beliefs about specific countries, the authors asked about a nonspecific hypothetical country in order to isolate the effect of democracy from any other opinions the respondent may have about a specific country. The challenge is when the characteristic of interest is changed (democracy), the respondents may also update their belief about related background characteristics, such as the hypothetical country’s GDP, dominant racial and religious groups, and region. For example, if you tell a respondent a hypothetical country is a democracy, they are more likely to assume the country has a high GDP, is majority Christian and white, and is located in Europe. This setting is an example of confounding: the treatment assignment is related to other variables which also influence the outcome. Thus, it is difficult to design an experiment that disentangles a respondent’s beliefs about democracy from their beliefs about other characteristics.

Dafoe, Zhang, and Caughey (2018) wanted to explore which experimental design would best isolate the democracy characteristics, and thus reduce confounding. They repeated Tomz and Weeks’s (2013) experiment using a series of different experimental designs and then used “placebo tests” to determine the best design. Their placebo test is a null contrast-control. They kept the primary active and control treatment levels (manipulating democracy vs. non-democracy), and chose alternative outcomes that *should* be unaffected by the treatment status in an *ideal* experiment: respondent’s assumptions about related characteristics like the country’s GDP and demographics. The authors found that a specific experimental design had the smallest effect on respondent’s assumptions about other characteristics of the hypothetical country.

##### Purpose of control:

Dafoe, Zhang, and Caughey (2018) used a null contrast-control in a pilot phase to design an experiment that minimized confounding.

## 5. Discussion and Practical Advice

We introduce controls defined on two different axes: null controls and non-null controls, and treatment, outcome, and contrast-controls. Null controls are related to potential outcomes or contrasts of potential outcomes which are *a priori* assumed to be zero; non-null controls are related to potential outcomes or contrasts of potential outcomes which are *a priori* assumed to be nonzero. A treatment-control is a treatment level with an assumed value for a potential outcome of interest. An outcome-control is an alternative potential outcome which has an assumed value given the active treatment level. A contrast-control is an alternative effect with an assumed value for the contrast between potential outcomes.

### 5.1. Discussion

As discussed in Section 1.2, our definitions relate to “negative control outcomes,” “negative control exposures,” “positive control outcomes,” and “positive control exposures” in previous statistical and epidemiological literature (Lipsitch, Tchetgen Tchetgen, and Cohen 2010; Tchetgen Tchetgen 2014; Shi, Miao, and Tchetgen Tchetgen 2020; Miao et al. 2024). All of these terms are specific cases of contrast-controls. If we choose the outcome to be the primary outcome of interest, $Y_{i}^{p}$, but choose alternative treatment levels, then we have a “negative control exposure”. If we choose the outcome to be a secondary outcome, but choose the primary active and control treatment levels of interest $w_{at}$ and $w_{ct}$, then we have a “negative control outcome”. We have introduced new terminology to make clear that this type of control is an assumption about an effect, which is a contrast between potential outcomes. An effect must always be estimated, and cannot be observed directly. Based on our understanding, treatment-controls and outcome-controls do not directly translate into this previous framework. We also depart from using the terms “negative” and “positive” to emphasize that the mathematical definitions do not correspond to positive or negative quantities. For example, a non-null treatment-control could result in an outcome that has a negative value if that corresponds to a non-null result.

If a control diagnostic fails, we have evidence that the experiment had a fundamental flaw. In contrast, if a control diagnostic does not fail, that does not prove that the experiment was not flawed. This idea is similar to the concept of a hypothesis test: we either reject the null hypothesis, or fail to reject it, but we do not accept the null hypothesis. Although this limitation is inherent to experimental controls, they are still valuable for checking the quality of an experiment. If we have an experiment with multiple well-chosen controls, we are more confident in the results. An alternative reason that a control diagnostic might fail is if the underlying scientific assumption was incorrect, rather than the experiment being flawed. Thus, controls should be carefully chosen using robust prior knowledge.

When designing an experiment, we recommend selecting a subset of controls that target the most likely points of error. If resources allow, we encourage experimenters to implement multiple types of controls, because different types are better suited to detecting different errors. The more complementary measurements other than our main quantities of interest, the more likely we are to detect any problems.

A complementary tool to controls is replicates, which increase the size or change the composition of the sample. Controls can help detect unwanted variation, which then can be addressed using a targeted set of replicates. The main sources of variation considered by biologists are biological and technical variation, which are explored using biological and technical replicates. Including biological replicates means repeating the experiment across different samples to account for “the intrinsic biological variability of a system” (Glass 2014, chap. 29). Technical replicates refers to repeating an experiment across independent experimental conditions to account for the variation independent of biological variability (Glass 2014, chap. 29). Choosing the right set of replicates helps explore the space of both wanted and unwanted variation.

We believe researchers should consider implementing either a pilot study or a pretrial period to collect detailed information on experimental controls. Collecting information about a wide variety of different controls can help the researcher better understand potential sources of variation and their magnitudes. Although this additional step is potentially costly, the extra step could ultimately lower costs if flaws in the design are identified in advance of the full experiment.

### 5.2. Practical Advice

Experimental controls can be useful tools in understanding different sources of variation in an experiment, either wanted or unwanted. For educators, introducing experimental controls can reinforce students’ understanding of fundamental concepts in statistics, such as estimands, confounding, and power. Some types of variation, in particular those that induce confoundedness of the treatment assignment, can result in a biased estimate of the causal effect. Other types can reduce power to detect causal effects.

Experimental controls must be tailored to the scientific context. One way to reinforce students’ understanding of experimental design is to given them real or hypothetical scenarios and ask them to identify which experimental controls would be most important and how to implement them. Experimental controls should target the most likely sources of unwanted variation. By identifying which controls to use, students are also identifying the weaknesses or limitations of an experiment. As we educate the next generation of researchers, teaching them about the effective use of experimental controls should lead to experiments that are high quality, transparent, and reproducible.

## Supplementary Material

Supp 1

The supplementary materials discuss other examples of using experimental controls during different experiment stages.

## Figures and Tables

**Table 1.** Control treatments.

Control treatment	Estimand: $Y\left(w_{at}\right)-Y\left(w_{ct}\right)$
$w_{\text{null}}$: No intervention	Effect of all components of coffee
$w_{ct1}$: Water	Effect of non-fluid component of coffee
$w_{ct2}$: Caffeinated water	Effect of non-caffeine component of coffee
$w_{ct3}$: Decaffeinated coffee	Effect of caffeine component of coffee

**Table 2.** Classifying controls.

	Null	Non-null
Treatment	null treatment-control $Y_{i}^{p}\left(w_{nt}\right)=0$	non-null treatment-control $Y_{i}^{p}\left(w_{nnt}\right)\neq 0$
Outcome	null outcome-control $Y_{i}^{no}\left(w_{at}\right)=0$	non-null outcome-control $Y_{i}^{nno}\left(w_{at}\right)\neq 0$
Contrast	null contrast-control $\tau_{i}^{nc}=0$	non-null contrast-control $\tau_{i}^{nnc}\neq 0$
