# Supplementary material for: Conceptualizing Experimental Controls Using the Potential Outcomes Framework
Source: Am Stat. Author manuscript; Available in PMC 2025 Nov 13. (PMC12609828; doi:10.1080/00031305.2025.2554756)
Supplement: Supp 1 [file NIHMS2118658-supplement-Supp_1.pdf]

Supplementary Material
Conceptualizing experimental controls using the potential outcomes framework

Kristen Hunter
University of New South Wales, Sydney, NSW, Australia
Kristen Koenig
Center for Systems Biology, Harvard University, Cambridge, MA, U.S.A.
and
Marie-Abèle Bind
MGH Biostatistics Center & Harvard Medical School, Boston, MA, U.S.A.

August 25, 2025

A Other uses of experimental controls in experimental design

A.1 Quality control for measurement error

Did our caffeine experiment have substantial measurement error? We discuss possible specific decision rules for quality control to determine if experimental results were subject to problematic levels of measurement error.

First, assume no measurement error in our primary outcome of change in blood pressure. Then, given our null treatment-control of no intervention, the primary outcome should be strictly zero for all subjects: $Y_i^p(w_{nt}) = 0$ for all i. However, blood pressure measurements are typically noisy. The observed outcome is the latent potential outcome with some added noise: $Y_i^{p,obs}\mathbb{1}(W_i = w_{nt}) = \mathbb{1}(W_i = w_{nt})Y_i^p(w_{nt}) + \epsilon_i$. Thus, even if w_{nt} is a valid null treatment-control, we could still see the observed outcome being nonzero, i.e., $\mid Y_i^{p,obs}\mathbb{1}(W_i = w_{nt}) \mid > 0$. We can choose different diagnostic statistics, and corresponding decision rules based on these statistics, to determine whether to reject an experiment depending on how strict we want our criteria to be.

Assuming a well-behaved and unimodal distribution of measurement error, we can choose simple diagnostic statistics. One statistic is the average change in blood pressure given the null treatment-control: ${N_{w_{n_t}}}^{-1} \sum_{i:W_i = w_{n_t}} Y_i^{p,obs}$. Based on our assumption that the null treatment-control results in a zero latent outcome, this number should be around zero. We can conduct a hypothesis test of $H_0 : N^{-1} \sum_{i=1}^{N} Y_i^p(w_{nt}) = 0$, and our decision

rule is we only accept the experiment as valid if this hypothesis test is not rejected. An alternative diagnostic statistic is the mean of the absolute values of the observed changes: ${N_{w_{n_t}}}^{-1} \sum_{i:W_i=w_{n_t}} \mid Y_i^{p,obs} \mid$. Alternatively, we can also choose a threshold A, and then define the statistic: the number of units where the absolute value of their outcome exceeds the threshold $N_A = \sum_{i:W_i=w_{n_t}} \mathbb{1}(\mid Y_i^{obs} \mathbb{1}(W_i = w_{nt}) \mid > A)$. Then, our decision rule is we require $n_A = 0$ for the experiment to be accepted. An example of a less strict rule is $n_A < N/10$, meaning no more than 10% of the population shows a large fluctuation in change in blood pressure.

The decision rule to reject the results of an experiment should be determined in advance by subject-matter experts, based on their understanding of the setting and how strict the researchers wish to be in quality control. For example, we may implement stricter rules for an experiment which is easily repeatable.

Implementing a control diagnostic decision rule results in some practical implications that are beyond the scope of this paper. Using multiple diagnostic tests could result in multiple testing problems. However, often the main concern with multiple testing is false positives. A false positive in a control test would result in discarding a legitimate experiment rather than the typical problem of reporting a spurious result. Additionally, power is an important consideration in diagnostic tests. For example, the hypothesis test for the null hypothesis $H_0 : \bar{Y}^p(w_{nt}) = 0$ could fail to be rejected because it is underpowered even if there is substantial measurement error. Smaller studies, while often more repeatable, are more likely to be underpowered. Ideally, planning for an appropriate sample size would also consider control diagnostic tests to ensure those tests are sufficiently powered.

The procedure we have described here applies to relatively simple systems, where we do expect measurement error, but we do not expect measurement error to change substantially across different experimental conditions. In this example, we do not expect measurement error to vary based on who is taking the blood pressure measurement or which device is being used to take the measurement. Thus, we propose a simple decision rule.

When a more complex variability structure is expected, a more structured approach to diagnosing measurement error would be recommended, such as a Gauge Repeatability and Reproducibility (Gauge R&R) study. In this context, reproducibility refers to variation across different experimental conditions, such as different operators or time periods (Burdick et al. 2005). Repeatability is variation for the same unit and same experimental conditions. A Gauge R&R study measures the level of variability due to measurement error, and evaluates the quality of the system. The design of the study can be complex, potentially including multiple factors that would result in different levels of measurement error and the interaction of these factors. A Gauge R&R study can be used in conjunction with a null treatment-control. The primary outcome given the null treatment-control is repeatedly measured across a variety of conditions, and then ANOVA can be used to break down the variability into different components and perform hypothesis tests.

A.2 Determining optimal timing

How long should we wait to measure blood pressure after giving subjects an intervention? In order to detect a caffeine effect, we must measure blood pressure during the time window

in which a subject is responding to caffeine. If we choose a time interval that is too long or too short, we will miss the effect and find a zero treatment effect of whether caffeine had any influence.

We can run a small pilot study to determine the best time window to detect caffeine effects for most people. One approach is to use a non-null treatment control, such as a hypertensive medication, and then measure blood pressure at many different points after the intervention. Although we might be tempted to run the pilot with the primary active treatment level, caffeinated coffee, we do not yet know whether caffeine affects blood pressure. The strategy of using the non-null treatment control assumes that the primary active treatment level (caffeinated coffee) and the non-null treatment control (hypertensive medication) operate on similar time scales. This assumption is likely plausible given that they are both orally administered and need to be metabolized into the bloodstream to produce physiological effects.

Another pilot study design is to use a non-null outcome control, such as reaction time. We would give subjects a cup of caffeinated coffee and then measure their reaction time at different time points to find the time of the peak effect. This second strategy assumes that the outcomes of reaction time and blood pressure respond on the same time scale. This assumption could be plausible given that once caffeine enters the blood stream, it has systemic effects. However, a subject-matter expert would need to judge whether this is a reasonable assumption, for example whether caffeine affects reaction time and blood pressure through the same mechanistic pathway.

When choosing between possible pilot designs, a main concern of the researcher may be cost and feasibility. Specific aspects of the experiment may be particularly difficult or costly, leading us to minimize the number of times we give a particular intervention or measure a certain outcome. For example, if the primary active treatment level is costly, we may choose a pilot design using a non-null treatment control. A non-null treatment control is necessarily a well-understood intervention, so it may be cheaper or have a smaller risk. For example, often in clinical trials the primary treatment is a novel medication and the non-null treatment-control is a standard treatment. Alternatively, the primary outcome may be difficult to measure, such as requiring expensive imaging. Then we may design a pilot using a non-null outcome control that is much easier to measure.

A.3 Identifying compliers and non-compliers

What if some subjects in our study do not comply with the given treatment? A complier is a subject who takes the treatment assigned to them, while a noncomplier does not. For example, a noncomplier is a subject who does not drink the coffee provided; perhaps they do not like the taste, and throw it away instead of consuming it. Compliance status can be measured during the main study. Alternatively, researchers could screen likely compliance status during a pre-trial period, in which advanced information is collected on the subjects that will be used in the main study.

We can measure a non-null outcome control during the pre-trial period to determine if people are likely to be compliers or noncompliers. We use change in electrolyte concentration as a non-null outcome-control because drinking any fluid (including coffee) changes

electrolyte concentration. If some units are given coffee but do not show a change in electrolyte concentration, they are likely noncompliers and we may choose to exclude them from the final study. In our caffeine study, noncompliance is probably unlikely, and may also not be particularly costly. However, in a large social science experiment implementing a costly intervention, a pre-trial step could potentially save substantial costs by ensuring the final study has a high proportion of compliers. If the complier average causal effect is the estimand of interest, pre-screening for compliance status allows the researcher to estimate this quantity with higher precision. For other estimands, excluding non-compliers may not be appropriate.

A.4 Identifying responders and non-responders

Do all the subjects in our study respond to caffeine? Population heterogeneity in unit-level causal effects is common, meaning different subjects respond differently to the same treatment levels. For example, we could have some units are responders, in that the primary treatment level has a nonzero effect on their outcomes, and other units are nonresponders, in that the primary treatment level has no effect or only a negligible effect on their outcomes.

In the caffeine example, some people in our population may be caffeine nonresponders, which we define as people who show no observable physiological responses from caffeine. Under this definition of a nonresponder, we are making a somewhat strict, but simplifying, assumption that that there are no partial responders. We assume that if someone doesn't show specific physiological responses to caffeine, they won't show any other potential responses either. We expect consistent responses across different types of physiological reactions, with no partial responders. During the pre-trial period we can use non-null contrast-controls, such as reaction time and alertness, to test for responder status. For each subject we measure reaction time with and without caffeine and estimate the causal effect. Then, we repeat the process with other non-null contrast-controls. If a subject shows no effect for these controls, we consider them a caffeine nonresponder according to our definition.

Our decision whether to include nonresponders in our study depends on our estimand of interest. If we are interested in a population-level estimand, we should include both nonresponders and responders. For example, a public health researcher deciding whether to implement a campaign aiming to reduce caffeine consumption would want the average effect of caffeine on blood pressure across the entire population. The population includes both responders and nonresponders, and that should be mirrored in our estimated causal effect.

If we are interested in unit-level causal estimands, we may want to exclude nonresponders from the study. For example, a doctor advising a patient with high blood pressure may want to know the worst-case scenario for the effect of caffeine on the patient's health. If we have limited resources, excluding nonresponders would increase the power to detect the effect of caffeine in responders, and the precision of the estimate. These issues are related to the idea of the generalizability of a study (Dahabreh et al. 2020). To avoid unconscious p-hacking, we recommend that the criteria for excluding non-responders be determined in advance.

References

Burdick, R. K., Borror, C. M. & Montgomery, D. C. (2005), *Design and Analysis of Gauge R&R Studies*, The American Statistical Association and the Society for Industrial and Applied Mathematics.

Dahabreh, I. J., Roberston, S. E., Steingrimsson, J. A., Stuart, E. A. & Hernán, M. A. (2020), 'Extending inferences from a randomized trial to a new target population', *Statistics in Medicine* **39**, 1999–2014.
